# Multiplex Detection of SNPs for Genetic Monitoring in Laboratory Mice by Luminex xTAG Assay

**DOI:** 10.3390/genes15121622

**Published:** 2024-12-19

**Authors:** Jiaqi Zhou, Jie Wei, Hong Wang, Huan Li, Lan Zhao, Rui Fu, Bingfei Yue

**Affiliations:** 1 Division of Laboratory Animal Monitoring, National Institutes for Food and Drug Control, Beijing 102629, China; jhan90657@gmail.com (J.Z.); weijie@nifdc.org.cn (J.W.); wanghong@nifdc.org.cn (H.W.); 13834661946@163.com (H.L.);; 2 R&D Center, Beijing Minhai Biotechnology Co., Ltd., Beijing 102629, China; 3 China National Rodent Laboratory Animal Resources Center, Beijing 102629, China

**Keywords:** SNPs, mice, inbred, outbred stock, genetic monitoring, Luminex xTAG assay

## Abstract

**Background:** The genetic quality of laboratory mice may have a direct impact on the results of research. Therefore, it is essential to improve genetic monitoring methods to guarantee research quality. However, few current methods boast high efficiency, high throughput, low cost, and general applicability at the same time. **Methods:** First, we got 34 SNP loci from previous studies for inbred strains and screened out 15 loci with good polymorphism for outbred groups from these 34 loci. Then, by using the Luminex xTAG assay, we tested inbred strains and outbred groups. **Results:** We tested commonly used inbred strains and five DNA samples from the International Council for Laboratory Animal Science, obtaining correct genotyping results. Additionally, some loci were potentially confirmed to be useful for distinguishing C57BL/6 and BALB/c mouse substrains. Furthermore, we tested three outbred groups and analyzed the genetic structure, and we compared the results of the SNP markers by xTAG assay to the STR markers by PCR, the trends of the three groups are the same. **Conclusions:** In our studies, the panels could meet the requirements for method promotion and provide a good choice for the genetic monitoring of inbred and outbred mice.

## 1. Introduction

Laboratory mice are commonly used in biomedical and behavioral research due to their significant benefits to human health [1,2]. Due to their low cost, small size, and availability, laboratory mice play an irreplaceable role in the field of biomedicine [3]. Many studies have attached great importance to genetic structural analysis and quality control to ensure the continued genetic diversity of a population [4,5,6].

The regular genetic quality monitoring of laboratory mice can provide reliable information and guidance for breeding and reproduction. Compared with outbred stocks, inbred strains are more stable, have less phenotypic variation, are better defined, are more uniform, have more extensive background data, and have a wider international distribution than outbred stocks [7,8]. The methods for breeding inbred strains and outbred stocks are completely different. Maintaining homogeneity of the genetic background is critical for breeding inbred strains, whereas heterozygosity is the principal emphasis in breeding outbred mice [9,10]. With the progress of molecular biology technology, DNA molecular markers have become the primary method of monitoring the genetic quality of laboratory mice. Most researchers have used microsatellite markers and single-nucleotide polymorphism (SNP) markers to detect genetic quality [4,11,12,13].

Various SNP genotyping techniques have been developed to improve detection accuracy, time, and price [14]. However, current SNP genotyping methods are characterized by high failure rates and low applicability [13,15,16]. Therefore, in the current study, we aimed to develop novel panels of SNP markers based on allele-specific primer extension (ASPE) and MagPlex-TAG microspheres. This could be used for genetic monitoring in inbred and outbred laboratory mice, which will lay the foundation for the genetic quality control of laboratory mice.

## 2. Materials and Methods

### 2.1. Animal Sample

Inbred mice were selected from Beijing and Shanghai for this study.

We collected 8 inbred strains from Beijing—C57BL/6JNifdc, C57BL/6JNifdc-bg, BALB/cJNifdc, BALB/cCrSlcNifdc, SCID/Nifdc, Nu/JNifdc, C3H/HeJNifdc, and DBA/2Nifdc—which were from the National Rodent Laboratory Animal Resources Center. The production license was SCXK (Jing)2022-0002.

We purchased C57BL/6, BALB/c, and NOD-SCID from Beijing HFK Bioscience Co., Ltd., Beijing, China, the production license was SCXK (Jing) 2019-0008.

We bought C57BL/6 and BALB/c from SPF (Beijing) Biotechnology Co., Ltd., Beijing, China, whose production license was SCXK (Jing) 2019-0010.

We procured the BALB/c from Beijing Vital River Laboratory Animal Technology Co., Ltd., Beijing, China, and the C57BL/6 from Peking University Health Science Center Department of Laboratory Animal Science. The production licenses were SCXK (Jing) 2021-0006 and SCXK (Jing) 2021-0013, respectively.

Three inbred strains, C57BL/6, BALB/c, and C3H/HeJ, were selected from National Rodent Laboratory Animal Resources, Shanghai Branch. The production license was SCXK (Hu)2017-0005.

Also, we obtained five DNA samples from the International Council for Laboratory Animal Science (ICLAS): C57BL/6J, C57BL/6NTac, BALB/cAnNTac, BALB/cJ, and C3H/HeNTac.

We obtained the KM/Nifdc, ICR/Nifdc, and NIH/Nifdc outbred groups from the National Rodent Laboratory Animal Resources Center were used, with 30 samples and half-sex from each strain.

All experiments followed the 3R principle and were approved by the Institutional Animal Care and the Committee of the National Institutes for Food and Drug Control 2022 (B)025.

### 2.2. SNP Locus Selection and Primer Design

We developed our approach based on previous reports [4] and information from the Central Institute for Experimental Animals (https://www.ciea.or.jp/en/, accessed on 1 March 2022). Using Primer Premier 6.0 software, we designed multiplex PCR primers and ASPE primers. An 801-base-pair (bp) template sequence, which included the SNP base and 400 bp of flanking regions, was input into the program. The 50 bp upstream and downstream of the SNPs were excluded from primer selection. The amplifications were designed to be relatively small with similar melting temperatures to facilitate efficient amplification in multiplex PCR [17,18]. All ASPE primers targeted both wild-type and mutant alleles through allele-specific nucleotides at the 3′ end and incorporated unique TAGs at the 5′ end, which were in reverse complement to the anti-TAGs on the xTAG beads.

### 2.3. DNA Extraction

The phenol–chloroform extraction method was used to extract DNA from tails [19]. All DNA concentrations were diluted to 50 ng/μL and stored at −20 °C.

### 2.4. Multiplex PCR Procedure and Agarose Gel Electrophoresis

The multiplex PCR was performed in a 30 μL reaction volume containing 15 μL of 2× Hotstart PCR Master Mix without dye by Sangon Biotech Co., Ltd., Shanghai, China, 10 μL of Sterilized ddH_2_O, 3 μL of a mixture of 10 pmol PCR primer, and 2 μL 50 ng of the extracted DNA template. The PCR protocol was as follows: a first extension at 96 °C for 30 s; a second at a suitable temperature for 1min; extensions at 72 °C for 30 s, 30 cycles; and a final extension at 72 °C for 8 min. All assays were run with positive and negative controls.

Amplified products were electrophoresed on a 2.5% agarose gel at 135 V for 35 min and stored at −20 °C for further analysis.

### 2.5. Exonuclease I-Shrimp Alkaline Phosphatase Treatment

After the multiplex PCR reaction, there are remaining amplified dNTPs, primers, single-stranded products, etc., which affect the subsequent reaction. Exonuclease I (Exo) was used to remove the remaining amplified primers and single-stranded products, and shrimp alkaline phosphatase (SAP) was used to remove the remaining primers, single-stranded DNA, and remaining dNTPs, especially dCTP, from the PCR products. Finally, 1 μL of Exo-SAP mixture was used to inactivate the enzyme, followed by a 30 min reaction at 37 °C and a 15 min reaction at 80 °C.

### 2.6. Allele-Specific Primer Extension (ASPE) Reaction

The total reaction system comprised 20 μL, containing 5 μL of purified multiplex PCR product, 1 μL of 10×Taq^TM^ Hot Start DNA polymerase buffer (Sangon Biotech Co., Ltd., Shanghai, China), 0.25 μL of 400 μmol/L Biotin-dCTP (Thermo Fisher Scientific, Waltham, MA, USA), 1 μL of 100 μmol/L dATP/ dGTP/dTTP mix(Thermo Fisher Scientific, Waltham, MA, USA), 0.15 μL of 5 U/μL Taq^TM^ Hot Start DNA polymerase (Sangon Biotech Co., Ltd., Shanghai, China), 1 μL of TAG-ASPE primer mix (containing 500 nmol/L of each TAG-ASPE primer), and 10.01 μL of dd H_2_O. This was mixed and centrifuged at a low speed (3000× *g*, 10 s), and the ASPE conditions was as follows: 94 °C for 30 s, 54 °C for 1 min, and 74 °C 2 min, 30 cycles.

### 2.7. Hybridization and Data Analysis

MagPlex-TAG microspheres were purchased from Luminex(Austin, TX, USA). Nucleic acid hybridization was performed in a 25 μL reaction volume containing 2.5 μL of ASPE reaction product and 22.5 μL of a working microsphere mixture, which included 2500 beads of each target-specific microsphere. The hybridization conditions were set at 96 °C for 90 s, 37 °C for 40 min. Subsequently, 100 μL of 1× SAPE buffer containing 8 μg/mL SAPE (Thermo Fisher Scientific, Waltham, MA, USA) was added, and the mixture was incubated at 37 °C for 20 min. The products were analyzed using the Luminex 200 detection system immediately after hybridization.

The Luminex 200 detection system (xPONENT3.1 software) provided median fluorescence intensity (MFI) values for all tested samples. Allelic MFI ratio = MFI_called allele_/(MFI_wild allele_ + MFI_mutant allele_). The result was performed according to the genotyping criteria provided by Luminex: MFI ratios above 0.75 or below 0.25 for wild-type or pure mutant type and between 0.25 and 0.75 for heterozygous mutant type.

For outbred groups, after we obtained the SNP genotyping results of 3 outbred groups, we used POPGENE 32 software version 1.0.0.0 [20] to calculate the observed number of alleles (Na), effective number of alleles (Ne), observed heterozygosity (Obs het), expected heterozygosity (Exp Het), average heterozygosity (Ave Het), and Shannon′s information index (SI); SNP loci were tested for polymorphism information content (PIC) by PIC-Calc 0.6 [21].

## 3. Results

### 3.1. SNP Locus Selection

We obtained 34 loci that could be stably amplified in inbred strains. We screened out 15 loci with good polymorphism for outbred groups from these 34 loci. Loci in these panels would be candidates for the final SNP marker evaluation systems. All SNP locus information is shown in Table 1.

Then, we randomly divided 34 loci into four panels [22]; the multiplex PCR primers are shown in Table 2(1)–(4), and the ASPE primers are shown in Table 3(1)–(4).

### 3.2. Result of Luminex xTAG Assay

Examples of the corrected data for the two inbred strains, BALB/c and C57BL/6J, used to develop this assay are shown in Table 4. SNPs were called when the corrected MFI was three times higher than the negative samples. In Table 4, the MFI values of the called alleles ranged from 1198.5 to 9184.5, while the MFI values of uncalled alleles ranged from 0 to 449.5. These results demonstrate that the Luminex xTAG Assay is a reliable tool for accurately identifying all targeted SNPs.

### 3.3. Analysis of Inbred Strains SNP Loci

We calculated the allele frequency for each strain based on the MFI detection results and determined the genotypes of each strain at 34 loci. The genotyping results showed that all strains were homozygous at all 34 loci, with no heterozygotes present. The specific genotyping results are shown in Table 5.

The genotyping results at the 34 SNP loci were used to compare the same strains from different rodent laboratory animal centers, and the comparison results are shown in Table 5(1).

Since C57BL/6 and BALB/c are the most widely used inbred mouse strains [23,24,25,26,27], we compared various sources of these strains, with the results shown in Table 5(2) and (3). From Table 5(2), it can be seen that the BALB/cJNifdc strain from BJ1 had different genotyping results at rs3712692, rs3706082, and rs3656801 compared to others.

Meanwhile, Table 5(3) reveals that the C57BL/6NTac strain exhibited differences at the loci rs3709624, rs3659787, rs3722313, rs3702158, and rs3724876 compared to C57BL/6 mice from other institutes. This suggests that C57BL/6NTac has diverged into substrains of C57BL/6, and these five loci can be used to differentiate between the substrains.

### 3.4. Population Genetic Structure Analysis

We obtained genetic data of three outbred groups at 15 loci. In KM mice, the observed number of alleles was 1.7333, the effective number of alleles was 1.4246, and the Shannon′s information index was 0.3749. Furthermore, the average heterozygosity was 0.2503. The average polymorphism information content (PIC) was 0.2006. The results are shown in Table 6(1).

In NIH mice, the observed number of alleles was 1.6667, the effective number of alleles was 1.2954, and the Shannon′s information index was 0.2829. Furthermore, the average heterozygosity was 0.1831 and the PIC was 0.1495. The specific data of the NIH group are shown in Table 6(2).

In ICR mice, the observed number of alleles, the effective number of alleles, the Shannon′s information index, the average heterozygosity, and the PIC were 1.7333, 1.3399, 0.3203, 0.2068, and 0.1688, respectively. The specific results are shown in Table 6(3).

### 3.5. Comparison of Sanger Sequencing and Luminex xTAG Assay

Currently, the most widely used SNP detection method is Sanger Sequencing (SS) [28]. Based on this, a comparison between SS and Luminex xTAG Assay is presented in Table 7.

## 4. Discussion

Laboratory mice play an irreplaceable role in the field of biomedicine. For their contributions to scientific and technological development, they are perceived as the touchstone and living precision instruments in research.

The stability of mouse quality is directly related to the repeatability of research results [29,30]. However, this is often overlooked, despite being a very important factor. Therefore, the genetic quality control of mice is the basic guarantee of scientific research quality.

Traditional methods such as biochemical markers include large sample consumption, time-consuming experimental procedures, and complex outcome discrimination. With the progress of molecular biology technology, one of the greatest improvements of molecular markers over biochemical markers is that laboratory animals no longer must be euthanized [9], and compared with Short Tandem Repeats (STRs), SNPs are more stable; the mutation rate is only 10^−9^. Furthermore, SNP detection requires lower sample quality than STRs. Since SNPs are biallelic markers, they can be detected automatically based on signals such as “+/−” or “1/0” [14,31]. This feature makes the SNP marker a better tool for achieving an automated and high-throughput analysis [14,32,33,34]. Based on the above advantages, the SNP marker-based assay was chosen in this study for laboratory mice.

Some platforms and methods exist for SNP-based genotyping, including ultra-high-throughput technologies like Illumina and Affymetrix. However, only a few of them are simultaneously flexible, fast (<1 day), cost-effective, and capable of simultaneously detecting multiplexed signals at medium-to high-throughput [35,36].

Luminex is a new type of biomolecular detection technology that can simultaneously detect multiple biomolecules such as antigens [37], antibodies [38], and nucleic acid [13] by flow cytometry [39]. It has emerged that Luminex not only offers the benefits of high throughput but also affords the advantages of high sensitivity, smaller sample volumes, and lower costs [40,41,42], which are suitable for multiple-sample analysis. The inside of the microsphere is stained with a precise number of two fluorescent agents of different spectra. This method allows for the simultaneous measurement of up to 100 different microspheres, each with a unique spectral address, in a reaction vessel. [17,38,43,44,45]. A fourth fluorescent pigment binds to the reporter molecule to detect ionic molecular interactions occurring on the surface of the microsphere. Each set of microspheres has multiple readings, providing valid and reliable statistics [46].

In this study, the method we established not only detected the international reference standards of ICLAS but also detected the commonly used strains of domestic rodent laboratory animal centers. The consistent genotyping results showed that the laboratory mice in various centers now have good genetic quality. Some strains have different genotyping results at individual loci; not only does this hint that these loci could indeed distinguish the substrains of BALB/c mice and C57BL/6 mice but it also suggests that we should pay more attention to and regularly monitor the genetic background of laboratory mice in different regions to ensure the reliability of our experimental results.

We calculated the average effective allele number, average effective heterozygosity, average Shannon′s index, PIC and others to evaluate genetic variation in three outbred groups. In addition, we compared the results of the SNP markers by xTAG assay to the STR markers by PCR, and it was found that whether using SNP markers or STR markers, the trends of the three groups are the same, namely KM > ICR > NIH. This result proves that our selected loci are very reliable for analysis in outbred groups. However, the number of SNP loci selected is small in this study, and the number of SNPs for screening outbred stocks should be expanded in subsequent studies to make the method more widely applicable to them.

## 5. Conclusions

In conclusion, the combination of SNP loci selected in this paper provides a good choice for the genetic monitoring of the quality and the population genetic structure of laboratory mice.

## Figures and Tables

**Table 1:** Number of alleles, location, and amplification primer sequences.

No.	SNP Loci	Chr.	No.	SNP Loci	Chr.
1 *	rs3022825	1	18	rs3023382	13
2 *	rs3022883	2	19	rs3091048	14
3 *	rs3022953	3	20	rs3091174	15
4 *	rs3022977	4	21 *	rs3023436	16
5 *	rs3023034	5	22	rs3023442	17
6	rs3023039	5	23 *	rs3023450	17
7	rs3023064	6	24 *	rs3089349	18
8	rs3023162	7	25	rs3023481	19
9 *	rs3023177	8	26 *	rs3089604	X
10 *	rs3023203	9	27	rs3709624	8
11 *	rs3023226	9	28	rs3659787	11
12 *	rs3089984	10	29	rs3722313	13
13	rs3089109	10	30	rs3702158	15
14 *	rs3088673	11	31	rs3724876	19
15 *	rs3023251	11	32	rs3712692	1
16	rs3023342	12	33	rs3706082	4
17	rs3023381	13	34	rs3656801	

Note: “*” indicates loci are screened for outbred group.

**Table 2:** (1) Panel A PCR primer sequences and amplification conditions; (2) Panel B PCR primer sequences and amplification conditions; (3) Panel C PCR primer sequences and amplification conditions; (4) Panel D PCR primer sequences and amplification conditions.

(1)			
SNP Loci	Product Size	Primer Sequences-F(5′~3′)	Primer Sequences-R(5′~3′)
rs3023381	169	GAGGTAGAGATGCAGCAGGG	GGAGGGATGGATGGTGGAGA
rs3023342	198	TGACTGGACCGTAACGAGAG	CAGGCCAGACCGTGCTAATG
rs3023481	250	TCTGGGCCACTACTTTCACTTC	CAGCACTTTCCACAGTTGGG
rs3089349	299	CCCTGTGCTCATGGTGTAGAAA	TCAGGGTGGATGATGTCCAAAG
rs3023382	337	CTAGGAGCCGCTGGTTCTGATAC	TGTGCTGCTTTCTGTGACATCTTC
rs3023162	378	TGTGTTCCCGTGACTGCTGAATG	GCCAGTACCAAGAGACCTCCCA
rs3023034	443	TGTGTAACTCTTGGCCCTTTCTGT	CCTGAATGGGCACTAGCACTCTA
rs3091174	495	GGCACTTCCCAACCAATCAACCA	ATGGAGGTTCATGCAACAGGTCAC
rs3091048	642	GATTCTGGAGGACCGCTTATCTGT	CCTCAAGCCGTTAGTGTCTCTACT
**(2)**			
**SNP Loci**	**Product Size**	**Primer Sequences-F(5′~3′)**	**Primer Sequences-R(5′~3′)**
rs3023251	170	TGGCAGGAGTGAATGAGTCAGAGG	AGTCAAAGTCTCCCTAGCTCTGGC
rs3088673	246	AACAAGACGGCACGGCTGAC	GCATGTCTCCTGATGGTGGAAGAT
rs3089604	290	TTTTCAGGGCCAAAGCAGACT	TCCTCCTAACTAAGTCCCCCTTTT
rs3089109	343	ACCAAGGTCTCTACCAAGCTCAA	TGTGAAATGGGTCTGTCAGAGTTT
rs3023203	388	TGGTCTCTTGGCATCTGGCATCT	ACAGAAACAAGAGCATCCGAAGCA
rs3023177	445	CCCATTGTGCGACTGTTCATTTGT	GAACTCCCTTCTGTGCCACTGTT
rs3022953	492	GAATGGTGAATGTCAGCTTCTGCT	CCTGTGCTACTCAGACCATGCTAT
rs3089984	590	ACTGGCGTGGTGTATGCGATTC	TCTCAGAGGAGGAGGTAGAGTTGG
**(3)**			
**SNP Loci**	**Product Size**	**Primer Sequences-F(5′~3′)**	**Primer Sequences-R(5′~3′)**
rs3023442	162	GAGACAGTTGAGGAGCGGAGGT	AGGGTGGAGACCGGAGATTTAGC
rs3023450	192	CTGACGTTCACCCTGCATCCTG	CCACACCTCCTTCTTGCTCACC
rs3023039	248	GCAAGAGACAAGCCAAGGCAGAT	GCCAACACTGGAAACAGCTAGGG
rs3023226	293	TGAGATCCAATGGCAAGGCACTAC	CAGGCTCTGGTTCACACTGATGT
rs3022977	338	CCTGTGGATTACTGTGCTGAAGAC	TTGCTCAACTATCAGGAATGTGCC
rs3023436	385	ACAGAATGCCTCTTTGACAATGGA	GGCAGTTGTACTTTCTGAAGAGTC
rs3022883	440	TCTCACCAGCACTGCCTCACT	TCCATCCTGTTTCTGGGTCTCTCA
rs3022825	506	AGCCAGCCATAGACCTGAAGTGT	GCCATCCGCCATTCCTCTAGTC
rs3023064	626	TTCCGCTATGTCTGACTGACTTGG	TCCTGTCTGTCTTTCTCTGTCTGC
**(4)**			
**SNP Loci**	**Product Size**	**Primer Sequences-F(5′~3′)**	**Primer Sequences-R(5′~3′)**
rs3724876	177	AAGTGTCAGTTTTATTGTTTCCAGT	CAAGTTCCCTTTTGGCTGGC
rs3702158	236	ACTCTCCAAAGAGGAACAGCTT	TACAGAGTACCCTAGCACGCC
rs3659787	277	CCACAACAAAGAGCCAGACCAA	TAGGCTTGGCCTTCATCTAAGC
rs3706082	358	CCACAGCGGGAATTGGATGGTATC	AGGCACAAGTGAGGCAGAAGAGA
rs3712692	411	AGTGAAGCTGAAATGTCTGGGAAA	AGAATGAGCCTGTGAAGTGAATGA
rs3722313	462	GGAAGCAGATGCGGAGGTGTAAG	TCAGGCTCTTGAGTAGCTGGACTT
rs3656801	525	AGCCATCTCTCCAGCCCAAGTC	GGACGTGTCACTCAGGGTGGAAA
rs3709624	619	GTCCTGTCGTCAGTGTATGAAGCA	GGGCTCAAATCCTCTTCTCCATCA

**Table 3:** (1) Panel A TAG-ASPE primer sequences; (2) Panel B TAG-ASPE primer sequences; (3) Panel C TAG-ASPE primer sequences; (4) Panel D TAG-ASPE primer sequences.

(1)			
SNP Loci	No.	TAG Sequences(5′~3′)	ASPE Primer(5′~3′)
rs3023381	19	ATACTTTACAAACAAATAACACAC	GGTCTCCCTGGTTCTCCTCG
	27	TAACTTACACTTAACTATCATCTT	GGTCTCCCTGGTTCTCCTCA
rs3023342	47	TCTCTTTAAACACATTCAACAATA	GCATAGACTCCAGATCCTTCT
	61	AATCTCTACAATTTCTCTCTAATA	GCATAGACTCCAGATCCTTCC
rs3023481	25	CTTTCTTAATACATTACAACATAC	GGAGACTATATATCTACTTTATGTGGAG
	53	TTAACAACTTATACAAACACAAAC	GGAGACTATATATCTACTTTATGTGGAA
rs3089349	58	CAATAAACATTCTTTACATTCTCA	TGGTAGTACTTATCTACCTTGG
	98	TTTACAACTACTAAACACACATTT	TGGTAGTACTTATCTACCTTGA
rs3023382	30	CTTAACATTTAACTTCTATAACAC	GTCTGCGACCTCAGGCAGAT
	94	ATACTAAACAATTCTCTTTACTAC	GTCTGCGACCTCAGGCAGAG
rs3023162	39	ACAAATATCTAACTACTATCACAA	GGTTCAAGGTATTCTTGTAGGG
	65	TACTTAAACATACAAACTTACTCA	GGTTCAAGGTATTCTTGTAGGA
rs3023034	89	TCACTTATTACTTCACATTATCTA	TTAAGGTAGAAAGCTAATAAAATAG
	96	CTAACTATAATCTTCATACACATA	TTAAGGTAGAAAGCTAATAAAATAT
rs3091174	15	TACTTCTTTACTACAATTTACAAC	TCCTTCTCCATCCCGCTCATCG
	73	CTTTATCAAATTCTAATTCTCAAC	TCCTTCTCCATCCCGCTCATCA
rs3091048	13	CAAATACATAATCTTACATTCACT	GCAAGTGTAGGAGGAGAGCTGT
	83	ACAATATACATCACTTAAACTTTC	GCAAGTGTAGGAGGAGAGCTGG
**(2)**			
**SNP Loci**	**No.**	**TAG Sequences(5′~3′)**	**ASPE Primer(5′~3′)**
rs3023251	19	ATACTTTACAAACAAATAACACAC	ATGAGTCAGAGGTCCTTCATTAGT
	39	ACAAATATCTAACTACTATCACAA	ATGAGTCAGAGGTCCTTCATTAGC
rs3088673	25	CTTTCTTAATACATTACAACATAC	AGATACAGCAGGGTGCATAT
	94	ATACTAAACAATTCTCTTTACTAC	AGATACAGCAGGGTGCATAG
rs3089604	61	AATCTCTACAATTTCTCTCTAATA	TGGCACTCAGTCACTGTTCT
	73	CTTTATCAAATTCTAATTCTCAAC	TGGCACTCAGTCACTGTTCC
rs3089109	65	TACTTAAACATACAAACTTACTCA	ATACATAGCTCAAGGAACATATAAAATATC
	83	ACAATATACATCACTTAAACTTTC	ATACATAGCTCAAGGAACATATAAAATATG
rs3023203	27	CTTTCTTAATACATTACAACATAC	AACTGACAATCAGGTAGACCATA
	58	CAATAAACATTCTTTACATTCTCA	AACTGACAATCAGGTAGACCATG
rs3023177	13	CAAATACATAATCTTACATTCACT	GTAGCCTTTCTAGTTCAGAACTTC
	89	TCACTTATTACTTCACATTATCTA	GTAGCCTTTCTAGTTCAGAACTTA
rs3022953	30	CTTAACATTTAACTTCTATAACAC	GTAACGGCAGTATTGAAAGACA
	53	TTAACAACTTATACAAACACAAAC	GTAACGGCAGTATTGAAAGACG
rs3089984	15	TACTTCTTTACTACAATTTACAAC	CCAGCTTTTCTGCAGTGATAGGAGA
	98	TTTACAACTACTAAACACACATTT	CCAGCTTTTCTGCAGTGATAGGAGC
**(3)**			
**SNP Loci**	**No.**	**TAG Sequences(5′~3′)**	**ASPE Primer(5′~3′)**
rs3023442	89	TCACTTATTACTTCACATTATCTA	GGGTTTCAGGTACCTCACA
	73	CTTTATCAAATTCTAATTCTCAAC	GGGTTTCAGGTACCTCACG
rs3023450	25	CTTTCTTAATACATTACAACATAC	CCACACCTCCTTCTTGCTCACC
	65	TACTTAAACATACAAACTTACTCA	CCACACCTCCTTCTTGCTCACT
rs3023039	30	CTTAACATTTAACTTCTATAACAC	ACCCCCACAGGAATCTCT
	61	AATCTCTACAATTTCTCTCTAATA	ACCCCCACAGGAATCTCC
rs3023226	15	TACTTCTTTACTACAATTTACAAC	GGAATATCGAGACATCACATAGCT
	19	ATACTTTACAAACAAATAACACAC	GGAATATCGAGACATCACATAGCC
rs3022977	27	TAACTTACACTTAACTATCATCTT	CCTTGAGGTCACCATGTCTCC
	94	ATACTAAACAATTCTCTTTACTAC	CCTTGAGGTCACCATGTCTCT
rs3023436	83	ACAATATACATCACTTAAACTTTC	CCAGGGAGTAAAACATCAGGA
	96	CTAACTATAATCTTCATACACATA	CCAGGGAGTAAAACATCAGGT
rs3022883	58	CAATAAACATTCTTTACATTCTCA	ATATTCAGAGAGTTAATTTCCTCTGCAGAA
	98	TTTACAACTACTAAACACACATTT	ATATTCAGAGAGTTAATTTCCTCTGCAGAC
rs3022825	39	ACAAATATCTAACTACTATCACAA	AGAGAATGTCAAGCCAGCTT
	53	TTAACAACTTATACAAACACAAAC	AGAGAATGTCAAGCCAGCTC
rs3023064	13	CAAATACATAATCTTACATTCACT	AAATAGGAACCAGAAGGAAACTGC
	47	TCTCTTTAAACACATTCAACAATA	AAATAGGAACCAGAAGGAAACTGA
**(4)**			
**SNP Loci**	**No.**	**TAG Sequences(5′~3′)**	**ASPE Primer(5′~3′)**
rs3724876	30	CTTAACATTTAACTTCTATAACAC	ATGTCAGGAGATCACCAGAGCG
	53	TTAACAACTTATACAAACACAAAC	ATGTCAGGAGATCACCAGAGCT
rs3702158	13	CAAATACATAATCTTACATTCACT	CTGAGAAAGGCCCAGTGTA
	39	ACAAATATCTAACTACTATCACAA	CTGAGAAAGGCCCAGTGTG
rs3659787	73	CTTTATCAAATTCTAATTCTCAAC	CTAGCGCACGAACTTACTAAG
	94	ATACTAAACAATTCTCTTTACTAC	CTAGCGCACGAACTTACTAAA
rs3706082	25	CTTTCTTAATACATTACAACATAC	CTAGGCCCTCTCAAGTTCCACCAT
	58	CAATAAACATTCTTTACATTCTCA	CTAGGCCCTCTCAAGTTCCACCAC
rs3712692	15	TACTTCTTTACTACAATTTACAAC	TTCCGTTTAAGCACTGATGTTTAAG
	61	AATCTCTACAATTTCTCTCTAATA	TTCCGTTTAAGCACTGATGTTTAAT
rs3722313	27	TAACTTACACTTAACTATCATCTT	CTGCCTTCTCCAGCTCCTTCCT
	65	TACTTAAACATACAAACTTACTCA	CTGCCTTCTCCAGCTCCTTCCC
rs3656801	89	TCACTTATTACTTCACATTATCTA	AGGCTCCCCTTAACTTATTGATG
	98	TTTACAACTACTAAACACACATTT	AGGCTCCCCTTAACTTATTGATA
rs3709624	19	ATACTTTACAAACAAATAACACAC	TGTAGTTCATTATCAACGAGAAGCT
	83	ACAATATACATCACTTAAACTTTC	TGTAGTTCATTATCAACGAGAAGCC

Note: The red bases are the identified SNP loci in the sequence.

**Table 4:** MFI values of each microsphere set corresponding to each allele of C57BL/6JNifdc and BALB/cJNifdc.

Panel No.	SNP Loci	C57BL/6JNifdc	BALB/cJNifdc
Panel A	rs3091048-T	2741	2926
	rs3091048-G	145	143.5
	rs3091174-G	2929	2668
	rs3091174-A	253.5	237
	rs3023034-G	1223	1131
	rs3023034-T	138	128
	rs3023162-G	2158.5	2124.5
	rs3023162-A	140	38
	rs3023382-T	6267	9
	rs3023382-G	43	2522
	rs3089349-G	1198.5	129
	rs3089349-A	131	498
	rs3023481-G	2613	107
	rs3023481-A	134	2411.5
	rs3023342-A	2801	220.5
	rs3023342-G	77.5	2803
Panel B	rs3089984-A	5406	43
	rs3089984-C	53	5205
	rs3022953-A	4628	4074.5
	rs3022953-G	135	82
	rs3023177-C	4548	0
	rs3023177-A	149	1490
	rs3023203-A	4806	4064
	rs3023203-G	91	175
	rs3089109-C	2402	1349
	rs3089109-G	0	171
	rs3089604-T	6379	6960
	rs3089604-C	24	328
	rs3088673-T	7404	63
	rs3088673-G	0	6686
	rs3023251-T	3667.5	67.5
	rs3023251-C	120	3542.5
	rs3089984-A	5406	43
	rs3089984-C	53	5205
Panel C	rs3023064-G	4009.5	175
	rs3023064-T	230	3405
	rs3022825-T	6557	5243.5
	rs3022825-C	380	117.5
	rs3022883-A	1795	1633.5
	rs3022883-C	117	35
	rs3023436-T	2764.5	67
	rs3023436-A	208	2311
	rs3022977-C	2319	183
	rs3022977-T	128.5	2359.5
	rs3023226-T	4027.5	309
	rs3023226-C	276.5	4220
	rs3023039-T	2645	2233.5
	rs3023039-C	257	0
	rs3023450-T	8656	385
	rs3023450-C	323	6832.5
	rs3023442-A	4574.5	4769.5
	rs3023442-G	393.5	449.5
Panel D	rs3709624-T	4728.5	0
	rs3709624-C	0	4470
	rs3659787-G	7870	74
	rs3659787-A	18.5	3273
	rs3722313-T	7518	110
	rs3722313-C	0	3273.5
	rs3702158-A	4990	0
	rs3702158-G	0	2785
	rs3724876-G	4096	0
	rs3724876-T	0	2120
	rs3712692-G	5109.5	0
	rs3712692-T	0	2200
	rs3706082-T	6029.5	0
	rs3706082-C	0	7479
	rs3656801-G	9184.5	92.5
	rs3656801-A	70	3971

**Table 5:** (1) The genotyping results of all samples; (2) The genotyping results of BALB/c samples; (3) The genotyping results of C57BL/6 samples.

(1)																																			
Regions	Strains	rs3022825	rs3022883	rs3022953	rs3022977	rs3023034	rs3023039	rs3023064	rs3023106	rs3023177	rs3023203	rs3023226	rs3089984	rs3089109	rs3088673	rs3023251	rs3023342	rs3023381	rs3023382	rs3091048	rs3091174	rs3023436	rs3023442	rs3023450	rs3089349	rs3023481	rs3089604	rs3709624	rs3659787	rs3722313	rs3702158	rs3724876	rs3712692	rs3706082	rs3656801
BJ11	C57BL/6JNifdc	T	A	A	C	G	T	G	G	C	A	T	A	C	T	T	A	G	T	T	G	T	A	T	G	G	T	T	G	T	A	G	G	T	G
	C57BL/6JNifdc-bg	T	A	A	C	G	T	G	G	C	A	T	A	C	T	T	A	G	T	T	G	T	A	C	G	G	T	C	G	T	A	T	G	T	G
	BALB/cJNifdc	T	A	A	T	G	T	T	G	A	A	C	C	C	G	C	G	A	G	T	G	A	A	C	A	A	T	C	A	C	G	T	T	C	A
	BALB/cAnSlacNifdc-nu	T	A	A	T	G	T	T	G	A	A	C	C	C	G	C	G	A	G	T	G	A	A	C	A	A	T	C	A	C	G	T	G	T	G
	BALB/cCrSlcNifdc	T	A	A	T	G	T	T	G	A	A	C	C	C	G	C	G	A	G	T	G	A	A	C	A	A	T	C	A	C	G	T	G	T	G
	SCID/Nifdc	T	A	A	T	G	T	T	G	A	A	C	C	C	G	C	G	A	G	T	G	A	A	C	A	A	T	C	A	C	G	T	G	T	G
	Nu/JNifdc	C	C	A	T	G	T	T	G	A	A	C	C	C	G	T	G	A	G	T	G	A	G	C	G	G	T	C	A	C	G	T	G	T	G
	C3H/HeJNifdc	C	C	G	T	T	T	T	G	A	A	C	A	C	G	C	G	A	G	T	A	T	G	C	G	G	T	C	A	C	G	T	G	T	G
	DBA/2Nifdc	C	C	G	T	T	C	T	G	A	A	C	A	C	T	C	G	A	T	T	A	T	A	C	G	G	C	C	A	C	G	T	G	T	G
BJ2	C57BL/6	T	A	A	C	G	T	G	G	C	A	T	A	C	T	T	A	G	T	T	G	T	A	T	G	G	T	T	G	T	A	G	G	T	G
BJ3	C57BL/6	T	A	A	C	G	T	G	G	C	A	T	A	C	T	T	A	G	T	T	G	T	A	T	G	G	T	T	G	T	A	G	G	T	G
BJ5	C57BL/6	T	A	A	C	G	T	G	G	C	A	T	A	C	T	T	A	G	T	T	G	T	A	T	G	G	T	T	G	T	A	G	G	T	G
BJ2	BALB/c	T	A	A	T	G	T	T	G	A	A	C	C	C	G	C	G	A	G	T	G	A	A	C	A	A	T	C	A	C	G	T	G	T	G
BJ3	BALB/c	T	A	A	T	G	T	T	G	A	A	C	C	C	G	C	G	A	G	T	G	A	A	C	A	A	T	C	A	C	G	T	G	T	G
BJ4	BALB/c	T	A	A	T	G	T	T	G	A	A	C	C	C	G	C	G	A	G	T	G	A	A	C	A	A	T	C	A	C	G	T	G	T	G
BJ2	NOD SCID	T	A	A	T	G	T	T	G	A	A	C	C	C	G	C	G	A	G	T	G	A	A	C	A	A	T	C	A	C	G	T	G	T	G
SH	C57BL/6	T	A	A	C	G	T	G	G	C	A	T	A	C	T	T	A	G	T	T	G	T	A	T	G	G	T	T	G	T	A	G	G	T	G
	BALB/c	T	A	A	T	G	T	T	G	A	A	C	C	C	G	C	G	A	G	T	G	A	A	C	A	A	T	C	A	C	G	T	G	T	G
	C3H/HeJ	C	C	G	T	T	T	T	G	A	A	C	A	C	G	C	G	A	G	T	A	T	G	C	G	G	T	C	A	C	G	T	G	T	G
ICLAS	C57BL/6J	T	A	A	C	G	T	G	G	C	A	T	A	C	T	T	A	G	T	T	G	T	A	T	G	G	T	T	G	T	A	G	G	T	G
	C57BL/6NTac	T	A	A	C	G	T	G	G	C	A	T	A	C	T	T	A	G	T	T	G	T	A	T	G	G	T	C	A	C	G	T	G	T	G
	BALB/cAnNTac	T	A	A	T	G	T	T	G	A	A	C	C	C	G	C	G	A	G	T	G	A	A	C	A	A	T	C	A	C	G	T	G	T	G
	BALB/cJ	T	A	A	T	G	T	T	G	A	A	C	C	C	G	C	G	A	G	T	G	A	A	C	A	A	T	C	A	C	G	T	G	T	G
	C3H/HeNTac	C	C	G	T	T	T	T	G	A	A	C	A	C	G	C	G	A	G	T	A	T	G	C	G	G	T	C	A	C	G	T	G	T	G
**(2)**																																			
**Regions**	**Strains**	**rs3022825**	**rs3022883**	**rs3022953**	**rs3022977**	**rs3023034**	**rs3023039**	**rs3023064**	**rs3023106**	**rs3023177**	**rs3023203**	**rs3023226**	**rs3089984**	**rs3089109**	**rs3088673**	**rs3023251**	**rs3023342**	**rs3023381**	**rs3023382**	**rs3091048**	**rs3091174**	**rs3023436**	**rs3023442**	**rs3023450**	**rs3089349**	**rs3023481**	**rs3089604**	**rs3709624**	**rs3659787**	**rs3722313**	**rs3702158**	**rs3724876**	**rs3712692**	**rs3706082**	**rs3656801**
BJ1	BALB/cJNifdc	T	A	A	T	G	T	T	G	A	A	C	C	C	G	C	G	A	G	T	G	A	A	C	A	A	T	C	A	C	G	T	T	C	A
BJ2	BALB/c	T	A	A	T	G	T	T	G	A	A	C	C	C	G	C	G	A	G	T	G	A	A	C	A	A	T	C	A	C	G	T	G	T	G
BJ3	BALB/c	T	A	A	T	G	T	T	G	A	A	C	C	C	G	C	G	A	G	T	G	A	A	C	A	A	T	C	A	C	G	T	G	T	G
BJ4	BALB/c	T	A	A	T	G	T	T	G	A	A	C	C	C	G	C	G	A	G	T	G	A	A	C	A	A	T	C	A	C	G	T	G	T	G
SH	BALB/c	T	A	A	T	G	T	T	G	A	A	C	C	C	G	C	G	A	G	T	G	A	A	C	A	A	T	C	A	C	G	T	G	T	G
ICLAS	BALB/cJ	T	A	A	T	G	T	T	G	A	A	C	C	C	G	C	G	A	G	T	G	A	A	C	A	A	T	C	A	C	G	T	G	T	G
**(3)**																																			
**Regions**	**Strains**	**rs3022825**	**rs3022883**	**rs3022953**	**rs3022977**	**rs3023034**	**rs3023039**	**rs3023064**	**rs3023106**	**rs3023177**	**rs3023203**	**rs3023226**	**rs3089984**	**rs3089109**	**rs3088673**	**rs3023251**	**rs3023342**	**rs3023381**	**rs3023382**	**rs3091048**	**rs3091174**	**rs3023436**	**rs3023442**	**rs3023450**	**rs3089349**	**rs3023481**	**rs3089604**	**rs3709624**	**rs3659787**	**rs3722313**	**rs3702158**	**rs3724876**	**rs3712692**	**rs3706082**	**rs3656801**
BJ1	C57BL/6JNifdc	T	A	A	C	G	T	G	G	C	A	T	A	C	T	T	A	G	T	T	G	T	A	T	G	G	T	T	G	T	A	G	G	T	G
BJ2	C57BL/6	T	A	A	C	G	T	G	G	C	A	T	A	C	T	T	A	G	T	T	G	T	A	T	G	G	T	T	G	T	A	G	G	T	G
BJ3	C57BL/6	T	A	A	C	G	T	G	G	C	A	T	A	C	T	T	A	G	T	T	G	T	A	T	G	G	T	T	G	T	A	G	G	T	G
BJ5	C57BL/6	T	A	A	C	G	T	G	G	C	A	T	A	C	T	T	A	G	T	T	G	T	A	T	G	G	T	T	G	T	A	G	G	T	G
SH	C57BL/6	T	A	A	C	G	T	G	G	C	A	T	A	C	T	T	A	G	T	T	G	T	A	T	G	G	T	T	G	T	A	G	G	T	G
ICLAS	C57BL/6	T	A	A	C	G	T	G	G	C	A	T	A	C	T	T	A	G	T	T	G	T	A	T	G	G	T	T	G	T	A	G	G	T	G
	C57BL/6NTac	T	A	A	C	G	T	G	G	C	A	T	A	C	T	T	A	G	T	T	G	T	A	T	G	G	T	C	A	C	G	T	G	T	G

Note: (1) BJ is Beijing; SH is Shanghai. (2) To facilitate base comparison, base T is colored in red, base A in green, and base C in blue.

**Table 6:** (1) The Na, Ne, Obs Het, Exp Het, Ave Het, SI, and PIC of the KM group; (2) The Na, Ne, Obs Het, Exp Het, Ave Het, SI, and PIC of the NIH group; (3) The Na, Ne, Obs Het, Exp Het, Ave Het, SI, and PIC of the ICR group.

(1)							
SNP ID	Na	Ne	Obs Het	Exp Het	Ave Het	SI	PIC
rs3023034	2	1.8	0.2222	0.4706	0.4444	0.6365	0.3457
rs3089349	2	1.5193	0.3125	0.3528	0.3418	0.5253	0.2834
rs3089984	2	2	1	1	0.5	0.6931	0.3750
rs3022953	2	1.1803	0.1667	0.1667	0.1528	0.2868	0.1411
rs3023177	1	1	0	0	0	0	0.0000
rs3023203	2	1.4562	0.1667	0.3222	0.3133	0.4926	0.2642
rs3089604	2	1.0377	0.037	0.037	0.0364	0.0922	0.0357
rs3088673	2	1.6653	0.4138	0.4065	0.3995	0.589	0.3197
rs3023251	2	1.8491	0.6429	0.4675	0.4592	0.6518	0.3537
rs3022825	2	1.9756	0.3704	0.5031	0.4938	0.687	0.3719
rs3022883	2	1.4824	0.2273	0.333	0.3254	0.5066	0.2724
rs3023436	2	1.4032	0.087	0.2937	0.2873	0.462	0.2460
rs3022977	1	1	0	0	0	0	0.0000
rs3023226	1	1	0	0	0	0	0.0000
rs3023450	1	1	0	0	0	0	0.0000
Mean	1.7333	1.4246	0.2431	0.2902	0.2503	0.3749	0.2006
St.Dev	0.4422	0.3613	0.2706	0.2645	0.1916	0.2709	0.1479
**(2)**							
**SNP ID**	**Na**	**Ne**	**Obs Het**	**Exp Het**	**Ave Het**	**SI**	**PIC**
rs3023034	1	1	0	0	0	0	0
rs3089349	2	1.6423	0.5333	0.4046	0.3911	0.5799	0.315
rs3089984	2	1.7041	0.25	0.4312	0.4132	0.6036	0.328
rs3022953	2	1.5571	0.3333	0.3701	0.3578	0.5433	0.294
rs3023177	1	1	0	0	0	0	0
rs3023203	2	1.5077	0.3333	0.3449	0.3367	0.5196	0.28
rs3089604	2	1.6964	0.2692	0.4186	0.4105	0.6008	0.326
rs3088673	2	1.0689	0.0667	0.0655	0.0644	0.1461	0.062
rs3023251	2	1.2677	0	0.2155	0.2112	0.3669	0.189
rs3022825	2	1.0425	0.0417	0.0417	0.0408	0.1013	0.04
rs3022883	2	1.8927	0.381	0.4832	0.4717	0.6645	0.36
rs3023436	2	1.0512	0.05	0.05	0.0488	0.1169	0.048
rs3022977	1	1	0	0	0	0	0
rs3023226	1	1	0	0	0	0	0
rs3023450	1	1	0	0	0	0	0
Mean	1.6667	1.2954	0.1506	0.1884	0.1831	0.2829	0.1495
St.Dev	0.4714	0.3194	0.1742	0.1892	0.1837	0.2643	0.1451
**(3)**							
**SNP ID**	**Na**	**Ne**	**Obs Het**	**Exp Het**	**Ave Het**	**SI**	**PIC**
rs3023034	2	1.1918	0.0588	0.1658	0.1609	0.2984	0.148
rs3089349	2	1.0377	0.037	0.037	0.0364	0.0922	0.036
rs3089984	1	1	0	0	0	0	0
rs3022953	1	1	0	0	0	0	0
rs3023177	2	1.5721	0.0435	0.372	0.3639	0.5501	0.298
rs3023203	2	1.0768	0.0741	0.0727	0.0713	0.1584	0.069
rs3089604	1	1	0	0	0	0	0
rs3088673	2	1.3846	0.3333	0.2825	0.2778	0.4506	0.239
rs3023251	2	1.9978	0.5667	0.5079	0.4994	0.6926	0.375
rs3022825	2	1.198	0.1818	0.1691	0.1653	0.3046	0.152
rs3022883	2	1.9716	0.4	0.5029	0.4928	0.6859	0.371
rs3023436	1	1	0	0	0	0	0
rs3022977	2	1.6	0.0455	0.3837	0.375	0.5623	0.305
rs3023226	2	1.28	0.1786	0.2227	0.2188	0.3768	0.195
rs3023450	2	1.7875	0.1034	0.4483	0.4405	0.6325	0.344
Mean	1.7333	1.3399	0.1348	0.2110	0.2068	0.3203	0.1688
St.Dev	0.4422	0.3487	0.1652	0.1865	0.1830	0.2570	0.1408

Note: Na = observed number of alleles; Ne = effective number of alleles; Obs Het = observed heterozygosity; Exp Het = expected heterozygosity; Ave Het = average heterozygosity; SI = Shannon′s information index.

**Table 7:** The comparison of Sanger Sequencing and Luminex xTAG Assay.

Feature	Sanger Sequencing	Luminex xTAG Assay
Throughput	Low throughput; only one locus can be detected at a time.	Higher throughput; efficient for multiplex detection.
Cost	Higher cost; one-panel samples need CNY 120.	Relatively lower cost; one-panel samples need CNY 30.
Data Analysis Complexity	Complex data analysis requires specialized software.	Relatively simple data analysis results are easy to interpret.
Time Efficiency	The corresponding samples need to be sent to a third party for testing.	Fast detection speed; results available in a short time (about 8 h).

## Data Availability

The data presented in this study are available on request from the corresponding author. The data are not publicly available due to privacy restrictions.
